# A Novel TLR4-Binding Domain of Peroxiredoxin From *Entamoeba histolytica* Triggers NLRP3 Inflammasome Activation in Macrophages

**DOI:** 10.3389/fimmu.2021.758451

**Published:** 2021-09-30

**Authors:** Xia Li, Meng Feng, Yanqing Zhao, Yuhan Zhang, Ruixue Zhou, Hang Zhou, Zhen Pang, Hiroshi Tachibana, Xunjia Cheng

**Affiliations:** ^1^ Department of Medical Microbiology and Parasitology, School of Basic Medical Sciences, Fudan University, Shanghai, China; ^2^ Department of Infectious Diseases, Tokai University School of Medicine, Isehara, Japan

**Keywords:** *Entamoeba histolytica*, NLRP3 inflammasome, peroxiredoxin, TLR4-binding domain, macrophage

## Abstract

Macrophages promote early host responses to infection by releasing pro-inflammatory cytokines, and they are crucial to combat amoebiasis, a disease affecting millions of people worldwide. Macrophages elicit pro-inflammatory responses following direct cell/cell interaction of *Entamoeba histolytica*, inducing NLRP3 inflammasome activation with high-output IL-1β/IL-18 secretion. Here, we found that trophozoites could upregulate peroxiredoxins (Prx) expression and abundantly secrete Prxs when encountering host cells. The C-terminal of Prx was identified as the key functional domain in promoting NLRP3 inflammasome activation, and a recombinant C-terminal domain could act directly on macrophage. The Prxs derived from *E. histolytica* triggered toll-like receptor 4-dependent activation of NLRP3 inflammasome in a cell/cell contact-independent manner. Through genetic, immunoblotting or pharmacological inhibition methods, NLRP3 inflammasome activation was induced through caspase-1-dependent canonical pathway. Our data suggest that *E. histolytica* Prxs had stable and durable cell/cell contact-independent effects on macrophages following abundantly secretion during invasion, and the C-terminal of Prx was responsible for activating NLRP3 inflammasome in macrophages. This new alternative pathway may represent a potential novel therapeutic approach for amoebiasis, a global threat to millions.

## Introduction

Human amoebic colitis and amoebic liver abscess (ALA) are caused by the protozoan parasite *Entamoeba histolytica*, which is estimated to affect 50 million individuals and lead to 40,000–100,000 deaths annually (1). Macrophages are crucial for resisting *E. histolytica* trophozoite from invading host tissues and organs. Studies based on animal models have indicated that ALA formation predominantly depends on the innate immune response of the host (2). Adequate responses induce the generation of large quantities of reactive oxygen species (ROS) and other effector molecules to eliminate *E. histolytica*, while inadequate responses give rise to ALA formation (2). As nitric oxide (NO), synthesized by inducible NO synthase (iNOS), is paramount in macrophage-mediated amoeba elimination, mice with iNOS deficiency were prone to ALA formation and hepatocyte apoptosis (3).

Inflammasome activation is one of the first innate immune events occurring during the host’s immune responses towards infection, which promotes the development of adaptive immune responses as well. Inflammasome activation involves pattern recognition receptors (PRRs) that sense pathogen-associated molecular patterns (PAMPs) or damage-associated molecular patterns (DAMPs) (4). Intracellular NOD-like receptor 3 (NLRP3) is widely known to be capable of recruiting apoptosis-associated speck-like proteins and pro-caspase-1 to form the NLRP3 inflammasome, thus inducing the maturation and secretion of interleukin (IL)-1β and IL-18 (5). Recently, several studies have indicated that *E. histolytica* is capable of rapidly activating inflammasomes in macrophages, and cysteine protease 5 (CP5) serves as the key component in NLRP3 inflammasome activation (6–8). Nevertheless, as *E. histolytica* can induce various patterns of inflammatory responses in the host, the other components in *E. histolytica* may also act as inducers of NLRP3 inflammasomes (3, 9).

Peroxiredoxin (Prx) is a ubiquitous and evolutionarily conserved family of peroxidase that efficiently and continuously reduces the production of hydrogen peroxide (H_2_O_2_), alkyl hydroperoxide, and peroxynitrite (ONOO^-^) during inflammation. Prxs can be divided into 1- and 2-cys Prxs according to the number of VCP motif-containing enzyme active centers present in the molecule (10). *E. histolytica* possesses more than 20 transcripts of Prxs, containing only the type of 2-cys. Among them, several types of 2-cys Prxs have been cloned and identified in laboratories (11–14). The H_2_O_2_-scavenging ability of Prxs decreases after removing its N-terminal, which contains the majority of cysteines (15). Due to the lack of catalase in *E. histolytica*, Prx is critical for scavenging H_2_O_2_ and protecting *E. histolytica* from damage caused by ROS that are released by phagocytes (16). Furthermore, Prx is expressed 50 times higher in *E. histolytica* than that in *Entamoeba dispar*, a nonpathogenic *Entamoeba* (17). Additionally, immunization with Prx can antagonize ALA formation in gerbils (18). Once *E. histolytica* adheres to and invades tissues and organs, the intracellular segment of Gal/GalNAc lectin recruits intracellular Prx to counteract oxidative damage (19). At present, functional studies on *E. histolytica* Prx predominately focus on its enzymatic activity and antigenicity, while the other functions were not reported. Prx is believed to have three main functions: protecting cells from damage caused by high concentrations of ROS/reactive nitrogen species; regulating redox pathways to control the synthesis and release of inflammatory mediators in inflammatory cells; acting as PAMPs or DAMPs to regulate inflammation formation through PRRs (10). Large amounts of Prxs are produced following parasitic invasion into tissues and organs (20). Prxs in *Plasmodium berghei* acts as PAMPs, binding toll-like receptor 4 (TLR4) on the surface of macrophages and promoting inflammation (21). Prxs in *Toxoplasma gondii* reduce the levels of ROS, caspase-1, and IL-1β in macrophages (22). Prxs in *Schistosoma mansoni* and *Fasciola hepatica* activate Th2 cells and macrophages, independent of their cysteine active sites (23). Therefore, the Prx in *E. histolytica* are speculated to interact with host macrophages as a PAMP molecule.

Here, we reveal that native Prx in *E. histolytica* (*Eh*-Prx) is involved in NLRP3 inflammasome activation in ALA mice model or macrophages. Prx serves as a PAMP molecule that induces NLRP3 inflammasome activation in macrophages through binding with TLR4 receptor and P2X purinoceptor 7 (P2X7) receptor, and its C-terminal is the key domain. Collectively, our study demonstrates that Prx in *E. histolytica* can activate NLRP3 inflammasome in a contact-independent manner.

## Materials and Methods

### Ethics Statement

All animal experiments were performed in strict accordance to the Regulations for the Administration of Affairs Concerning Experimental Animals (1988.11.1), and approved by the Institutional Animal Care and Use Committee (IACUC) of our institutions (Permit Numbers: 20160225-097). All efforts were made to minimize animal suffering.

### Parasite and Cell Culture

*E. histolytica* SAW755CR and HM-1:IMSS strains were grown axenically in YIMDHA-S medium containing 15% and 10% (v/v) heat-inactivated bovine serum at 36.5°C, respectively. RAW264.7 cells were cultured in Dulbecco’s modified Eagle medium (Gibco, USA) with 10% (v/v) fetal bovine serum (FBS) (Thermo Fisher, USA), 100 U/ml of penicillin (Gibco, USA), and 100 μg/mL of streptomycin (Gibco, USA). CHO-K1 cells were cultured in Ham’s F12 medium (Corning, USA) with the same concentrations of FBS, penicillin, and streptomycin.

### ALA Animal Model

Ten- to twelve-week-old male C57BL/6 mice were purchased from Shanghai Slake Laboratory Animal Company, China. ALA was subsequently induced in the mice by inoculating 10^6^ SAW755CR trophozoites under liver capsule. Five days later, the liver tissue was immediately collected for histopathology and immunohistochemical staining, or frozen at -80°C for qPCR analysis. Sections of ALA tissue or normal liver tissue were stained with HE or PAS. Meanwhile, anti-F4/80 antibody (Cell Signaling Technology, USA), *Eh*-Prx 4G6 monoclonal antibody (mAb) (24), and anti-IL-1β antibody (Cell Signaling Technology, USA) were used for detecting the distribution of macrophages, *E. histolytica*, and IL-1β, respectively. High-quality pictures were captured by Nikon ECLIPSE Ci-L microscope (Olympus, Japan).

### Quantitative Analysis of Related Protein Gene Expression Levels by qPCR

Total RNA was isolated using the RNeasy Plus Mini kit (Qiagen, Germany). cDNA was synthesized using the Primescript first-strand DNA synthesis kit (Takara #6110A) with primers listed in **Table 1** or oligo (dT). qPCR was performed on an ABI 7500 Real-time PCR System (Applied Biosystems, USA) using TB Green Premix Ex Taq (Takara, RR420A).

**Table 1:** Primers used in this study.

Primers	Sequence
*Eh-Prx* forward	CCCATATGTCTTGCAATCAACAAAAAGAGT
*Eh-Prx* reverse	CCGGATCCTTTTAATGTGCTGTTAAATATT
*Eh-Prx*-Fragment-1 forward	CCCATATGTCTTGCAATCAACAAAAAGAGT
*Eh-Prx*-Fragment-1reverse	CCGGATCCTTTTATTGTCCTGCAAGTTCACTAT
*Eh-Prx*-Fragment-2 forward	CCCATATGGGAAAATATGTTGTATTGTTGTTT
*Eh-Prx*-Fragment-2 reverse	CCGGATCCTTTTAATCATCAATGATGACATATC
*Eh-Prx*-Fragment-3 forward	CCCATATGAAGTTGACATTCCCATTAGTATCA
*Eh-Prx*-Fragment-3 reverse	CCGGATCCTTTTAATGTGCTGTTAAATATT
*Eh-Prx*-cys1-C2G forward	GGATTGGACATTTGTTGGTCCAACAGAAATGATTGG
*Eh-Prx*-cys1-C2G reverse	CCAATCATTTCTGTTGGACCAACAAATGTCCAATCC
*Eh-Prx*-cys2-C2G forward	GAACATGGAGCAGTTGGTCCACTCAATTGGAAAC
*Eh-Prx*-cys2-C2G reverse	GTTTCCAATTGAGTGGACCAACTGCTCCATGTTC
Mouse-*β-actin* forward	TCTACAACGAGCTGCG
Mouse-*β-actin* reverse	CAATTTCCCTCTCGGC
Mouse-*NLRP3* forward	TTGAAGAAGAGTGGATGGGTTTGC
Mouse-*NLRP3* reverse	GCGTTCCTGTCCTTGATAGAGTAG
Mouse-*caspase-11* forward	ACAATGCTGAACGCAGTGAC
Mouse-*caspase-11* reverse	CTGGTTCCTCCATTTCCAGA
Mouse-*caspase-1* forward	GAAGAACAGAACAAAGAAGATGGCACA
Mouse-*caspase-1* reverse	AGCTCCAACCCTCGGAGAAAGAT
Mouse-*IL-1β* forward	GCTTCAGGCAGGCAGTATCACTC
Mouse-*IL-1β* reverse	GTGCAGTTGTCTAATGGGAACGT
Mouse-*IL-18* forward	CATGTCAGAAGACTCTTGCGTCAA
Mouse-*IL-18* reverse	TTTATATTCCGTATTACTGCGGTTGT
Reverse Transcription (RT)- Mouse-*NLRP3*	TCGGCAGTGGATAAAGAACAAATAGTC
RT- Mouse-*caspase-11*	ATCAATGGTGGGCATCTGGGAAT
RT- Mouse-*IL-1β*	TCTGCTTGTGAGGTGCTGATGTA
RT- Mouse-*IL-18*	AGAGTGAACATTACAGATTTATCCC
RT- Mouse-*β-actin*	TCTGCTGGAAGGTGGACAGTGAG

### Detection of Prx Localization in Trophozoites by Laser Confocal Microscopy

A total of 10^6^ RAW264.7 or CHO-K1 cells were cultured in a 35 mm cell culture dish (Corning, USA) with sterilized cover slides (22 × 22 mm). Once the cells completely adhered to the slide, 10^6^ trophozoites were added to the culture dish. After incubating at 36.5°C for 1 h, the coverslips were fixed and incubated with 0.01% Triton X-100. After blocking, trophozoites were then incubated with mAb 4G6, followed by Alexa Fluor 488-labeled goat anti-mouse IgG (H+L) (Invitrogen, USA). Slides were observed under a laser confocal microscope (Leica, SP8, Germany).

### Detection of Prx Secretion by Western Blotting

A total of 5×10^5^ RAW264.7 or CHO-K1 cells were cultured in a 24-well plate. Once the cells completely adhered to the slide, 5×10^5^ trophozoites were added to the wells. After incubating at 36.5°C for 1 h in relatively anaerobic environment, the culture supernatant was collected. Then, cold acetone precipitation method was applied to extract protein from the culture supernatant. The extracted protein was resuspended in 30 μL loading buffer (containing β-ME). Trophozoite lysate was used as the positive control. The protein samples were separated by 10% SDS-PAGE and detected by western blotting. 4G6 (mouse ascites, 1:400), and Goat anti mouse IgG (H + L) (Abcam, UK) were used as the primary and secondary antibodies, respectively.

### Purification of *E. histolytica* Prxs Using Affinity Chromatography With mAb 4G6

An aliquot of 7.5 mg mAb 4G6 was coupled with pretreated cyanogen bromide (CNBr)-activated agarose gel (Pharmacia Biotech, 17-0430-01, USA) according to the manufacturer’s instruction. Then, trophozoites were resuspended with solubilization buffer. Following sonication, the solubilized trophozoites were centrifuged and filtered through a 0.2 μm filter, mixed with resin, and incubated at 4°C for 2 h. The bound protein was eluted using elution buffer. After dialysis, the protein solution was immediately filtered through a 0.2μm filter for sterilization. RAW264.7 cells were pretreated with TAK242 (1 μM) and then incubated with native Prx (5 μg/mL) for 6 h.

### *E. histolytica* Recombinant Protein Expression and Identification

Prx (XP_648522.1) and its three fragments (Fragment-1: from 1 to 100 amino acids; Fragment-2: from 71 to 170 amino acids; Fragment-3: from 134 to 233 amino acids) were amplified from *E. histolytica* cDNA using primers listed in **Table 1**. The gene fragment was inserted into a pET19b vector (Novagen, Germany) and transformed into *E. coli* BL21 (DE3) pLysS for protein expression. Mutant Prx was also cloned using primers listed in **Table 1**. Based on nucleotide sequence of *Eh*-Prx (XM_643430.2), the codon of cysteine in both cysteine active sites of *Eh*-Prx (from 5’ to 3’, at sites of 264 and 627, respectively) was mutated from UGU to GGU, resulting in mutation from cysteine to glycine. Recombinant protein was filtered through a 0.2 μm filter, while endotoxin levels of the recombinant protein were detected using a Toxin Sensor™ Gel Clot Endotoxin Assay Kit (Genscript, L00351, USA).

### Construction of Peritonitis Mice Model to Detect the Expression Levels of Related Proteins by qPCR

Ten- to twelve-week-old female C57BL/6 mice were purchased from the Shanghai Slake Laboratory Animal Company and intraperitoneally injected with or without *Eh*-rPrx (100 μg). Twelve hours later, following sacrificing under anesthesia, the mice were intraperitoneally injected with 5 mL of cold PBS and gently massaged for 5 min. The cells were collected as peritoneal exudate cells (PEC), which partly composing of macrophages. Total RNA and cDNA were obtained using the methods described above, and qPCR primers used are listed in **Table 1**.

### Detection of NLRP3 Activation by Western Blotting

Following co-incubating native Prx or *Eh*-rPrx with RAW264.7 cells for 6 h, the cell lysate was collected for western blotting analysis. Primary antibodies used in this experiment included anti-β-actin (Abcam, UK), anti-NLRP3 (Cell Signaling Technology, USA), and anti-caspase-11 (Abcam, UK) antibodies; the secondary antibody used in this experiment was horseradish peroxidase-goat anti-rabbit IgG (H+L) (Abcam, UK). After co-incubating *Eh*-rPrx and RAW264.7 cells for 24 h, cell supernatant was collected. Similarly, cold acetone precipitation method was applied for protein extraction. The primary antibody used for western blotting was cleaved caspase-1 (Asp296) monoclonal antibody (Cell Signaling Technology, USA). ImageJ 1.52a (Wayne Rasband National Institutes of Health, Bethesda, MD, USA) was used for evaluating signal intensity.

### Detection of Cytokine Secretion in Cell Culture by ELISA

After co-incubating *Eh*-rPrx with RAW264.7 cells for 12 and 24 h, cell supernatant was collected, and the levels of IL-1β and IL-18 were detected using an IL-1β kit (R&D Systems, MLB00C, USA) and an IL-18 kit (Abcam, UK), respectively.

### siRNA Treatment on RAW264.7 Cells

TLR4 siRNA (CAATTCTGTTGCTTGTATA) and control siRNA (targeting β-actin) were synthesized by Guangzhou RiboBio Co., Ltd, China. RAW264.7 cells were cultured in a 24-well plate and siRNA (50 nM) was added by Lipofectamine^®^ RNAiMAX Reagent Kit (Invitrogen, USA). After 24 h, the cells were incubated with Tris-HCl (pH 8.0) or *Eh*-rPrx (5 μg/mL).

### Detection of *Eh*-rPrx-Induced Cytotoxicity

*Eh*-rPrx-induced cytotoxicity was detected using an Annexin V-FITC Apoptosis Detection Kit (Sigma-Aldrich, APOAF, USA). Following incubation with *Eh*-rPrx for 24 h, the RAW264.7 cells were collected and detected according to the manufacturer’s instruction. Fluorescence of the cells was immediately determined using a flow cytometer (FACSCalibur, B-D, USA).

### Mitochondrial ROS Observation and Quantification by High-Content Screening Analysis

After co-incubating *Eh*-rPrx with RAW264.7 cells for 24 h and 36 h, the cells were washed with warm HBSS/Ca/Mg (Gibco, Germany) and stained with 5 μM MitoSOX™ reagent (Invitrogen, USA). CFSE (2 μM) was used for counterstaining. The cells were kept in warm buffer, followed by immediate observation and quantification through high-content screening analysis (Perkin-Elmer, Operetta, USA).

### Statistical Analyses

Data are presented as mean ± SEM. Statistical analyses were performed using GraphPad Prism 5 (GraphPad Software, Version 5.01, USA). Student’s *t* test was performed for comparison between the control and treatment groups. A p value of < 0.05 was considered significant for all analyses.

## Results

### Expression of *E. histolytica* Prx and Priming of NLRP3 Inflammasome in ALA Tissue

**Figure 1:**
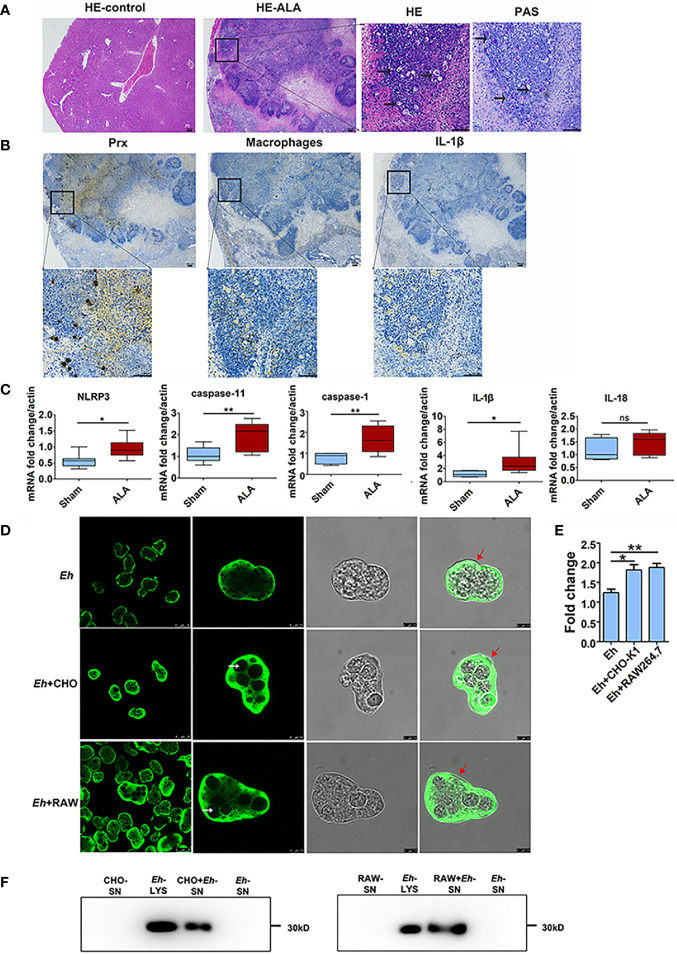
Priming of NLRP3 inflammasome in ALA tissue and Secretion of Prx in *E histolytica*. **(A)** Construction of mice ALA model. Male C57BL/6 mice were inoculated with 1 × 10^6^ trophozoites of the *E histolytica* SAW755CR strain/mouse. Five days after treatment, the ALA tissue was obtained. Sections from normal or ALA tissue were observed by HE and PAS staining. Bars: 100 μm. Black arrows: trophozoites. Experiments were repeated twice. **(B)** Immunohistochemical staining analysis of ALA. Sections of ALA tissue were incubated with anti-F4/80 antibody, mAb 4G6, or anti-IL-1β antibody to detect distribution of macrophages, Prx of *E histolytica*, or IL-1β in ALA tissue, respectively. Brown areas were considered positive. Bars: 100μm. **(C)** Expression of NLRP3 inflammasome-related genes in ALA tissue. Male C57BL/6 mice were inoculated with 10^6^ trophozoites of the *E histolytica* SAW755CR strain. Five days after infection, the expression levels of related genes in ALA tissue were measured through qPCR, and shown as 2-ΔΔCt of the target gene relative to β-actin, normalized with corresponding values in normal mice. In sham group, n = 6; in ALA group, n = 8. Experiments were repeated twice. **(D)** The location of Prx in trophozoites co-incubated with CHO-K1 or RAW264.7 cells. Trophozoites during the logarithmic growth phase were harvested and co-incubated with CHO-K1 or RAW264.7 cells. An hour after incubation, an *E histolytica* 4G6 monoclonal antibody (mAb) was used to measure the expression level and localization of Prx through immunofluorescence assay and laser confocal microscopy. White arrows: engulfed cells; red arrows: trophozoites membrane. Bars, 25 μm (the left column) or 7.5 μm (right three columns). **(E)** Quantitative analysis of Prx fluorescence intensity of *E histolytica*. Cells were observed with the same laser intensity by laser confocal microscopy. The fluorescence intensity of Prx was analyzed using ImageJ 1.52a. In all groups, n = 4. Experiments were repeated three times. **(F)** Prx secretion from *E histolytica* trophozoites was detected by western blotting. After incubated with CHO-K1 or RAW264.7 cells in serum-free cell medium under anaerobic condition, cell supernatant was collected. Cold acetone precipitation was conducted to extract protein from culture supernatant. Afterwards, protein from the same volume of supernatant was analyzed by western blotting. Experiments were repeated twice. CHO, CHO-K1 cells; RAW, RAW264.7 cells; SN, supernatant; Lys, lysate. Statistical analysis was conducted by Student’s *t*-test, and data are presented as mean ± standard error of mean (SEM). *p < 0.05, **p < 0.01, ns, not significant.

An ALA model was constructed in C57BL/6 mice. Five days after inoculating *E. histolytica* SAW755CR trophozoites into the liver, an abscess was observed through hematoxylin and eosin (HE), with several trophozoites on the edge of abscess when stained with periodic acid-Schiff (PAS) (**Figure 1A**).

Furthermore, we detected the distribution of macrophages and *Eh*-Prx by immunohistochemical staining (**Figure 1B**). The results showed that a large number of macrophages were distributed on the edge of the abscess, and *Eh*-Prx was detected not only on the edge but also in the center of the abscess. Interestingly, expression of Prx and the presence of macrophages were found in similar areas, suggesting the potential interaction between macrophages and *Eh*-Prx. In addition, the secreted IL-1β was also detected in the same area by immunohistochemical staining (**Figure 1B**).

Real-time quantitative polymerase chain reaction (qPCR) was conducted to detect expression levels of NLRP3 inflammasome-related genes in liver abscess tissue (**Figure 1C**). Compared with the control group, the gene expression levels of *NLRP3*, caspase-1, and *IL-1β* were significantly higher in the experimental group (p < 0.05), while *IL-18* was up-regulated slightly, suggesting priming of the NLRP3 inflammasome in ALA tissue. Besides, the key component of non-canonical activation pathway for NLRP3 inflammasome, *caspase-11*, was also up-regulated in ALA tissue.

### Secretion of Prx by *E. histolytica* Encountering Host Cells

To further explore the dynamic changes of localization and secretion of Prx by *E. histolytica* following the invading tissues and organs, trophozoites were incubated with CHO-K1 and RAW264.7 cells, respectively. The results showed that after incubation with CHO-K1 cells for 1 h, the trophozoites engulfed several cells (**Figure 1D**). Compared with the control group, aggregated Prx was observed both in submembrane and cytoplasm (**Figure 1D**), and the fluorescence intensity of Prx in trophozoites was significantly increased (**Figure 1E**). After co-incubating trophozoites with RAW264.7 cells, the localization and fluorescence intensity of Prx were consistently changed as those observed in CHO-K1 cells (**Figures 1D, E**). The trophozoites alone did not secrete Prx at anaerobic environment, while they secreted a large amount of Prx once encountering with CHO-K1 or RAW264.7 cells (**Figure 1F**), suggesting that Prx might be involved in the pathogenesis of trophozoites.

### Priming of NLRP3 Inflammasome in Macrophages by Native Prx

**Figure 2:**
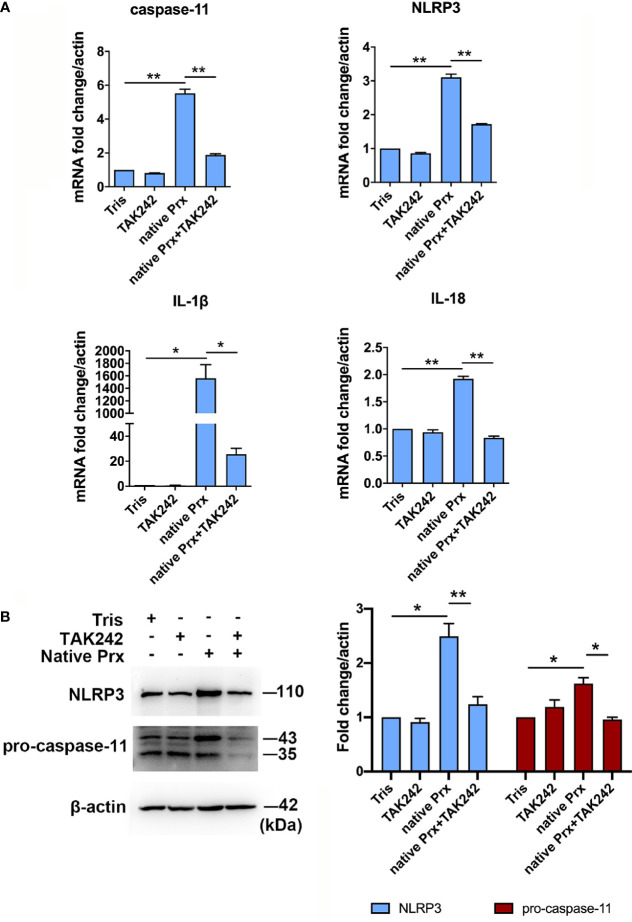
Priming of NLRP3 inflammasome in macrophages by native Prx. **(A)** Gene expression levels detected by qPCR. RAW264.7 cells were treated with 5 μg/mL of native Prx, while LPS (1 μg/ml) and Tris-HCl (pH 8.0) was used as the control. Six hours after the treatment, the expression levels of related genes were measured by qPCR, and shown as 2-ΔΔCt of the target gene relative to β-actin, normalized with corresponding values. In all groups, n = 3. Experiments were repeated three times. **(B)** Western blot analysis of NLRP3 inflammasome-related proteins. RAW264.7 cells were pretreated with TAK242 (1 μM) for 1 h. Next, the cells were treated with either 5 μg/mL of native Prx or Tris-HCl (pH 8.0). Six hours after the treatment, the cells were collected, and the cell lysis was analyzed by western blotting. In all groups, n = 3. Experiments were repeated three times. Statistical analysis was conducted by Student’s *t*-test, and data are expressed as mean ± SEM. *p < 0.05, **p < 0.01.

Native Prxs were collected from the lysate of *E. histolytica* trophozoites and identified using an mAb 4G6 (**Supplementary Figure 1**). They were composed of a mixture of seven Prxs based on Mass Spectrometry, including the Prx that was cloned and expressed in our previous work (XP_648522.1) (16) (**Supplementary Table 1**). The protein sequence alignment *via* DNAMAN 6.0 showed that the peptide length of these seven Prxs was 229 to 237 amino acids, while all of them belonged to 2-cys Prx. The amino acid residue consistency of these Prxs was as high as 97.54% (**Supplementary Figure 2**), indicating their similar functions. After incubating the native Prxs with RAW264.7 cells for 6 h, the gene and protein expression levels of NLRP3 inflammasome related proteins were detected by qPCR and WB, respectively. The results showed that *NLRP3*, *IL-1β*, *IL-18*, and *caspase-11* were significantly upregulated by the native Prxs (**Figures 2A, B**), which was dramatically reduced by TLR4 receptor inhibitor TAK242. It suggested that NLRP3 inflammasome was primed by native Prx of *E. histolytica via* TLR4 receptor.

### Priming of NLRP3 Inflammasome in PEC by *Eh*-rPrx

**Figure 3:**
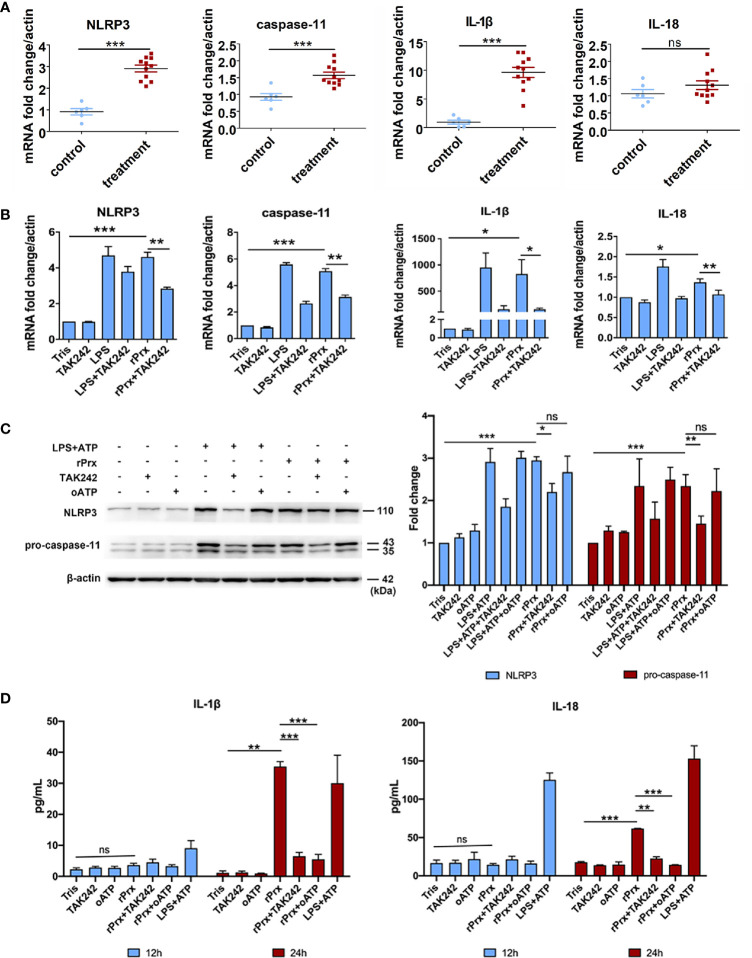
*Eh*-rPrx-Activation of NLRP3 Inflammasome in RAW264.7 Cells. **(A)** Female C57BL/6 mice were treated by intraperitoneal injection of *Eh*-rPrx (100 μg/mouse); Tris-HCl (pH 8.0) was used as the negative control. Twelve hours after the treatment, PEC (containing macrophages) were collected, and the expression levels of related genes were measured by qPCR and shown as 2-ΔΔCt of the target gene relative to β-actin, normalized with corresponding values in the negative control. In control group, n = 6; in treatment group, n = 11. Experiments were repeated twice. **(B)** Gene expression levels detected by qPCR. RAW264.7 cells were treated with 5 μg/mL of *Eh*-rPrx, while LPS (1 μg/ml) and Tris-HCl (pH 8.0) was used as the control. Six hours after the treatment, the expression levels of related genes were measured by qPCR, and shown as 2-ΔΔCt of the target gene relative to β-actin, normalized with corresponding values. In all groups, n = 3. Experiments were repeated three times. **(C)** Western blot analysis of NLRP3 inflammasome-related proteins. RAW264.7 cells were pretreated with TAK242 (1 μM) for 1 h or oATP (500 μM) for 2 h. Next, the cells were treated with either 5 μg/mL of *Eh*-rPrx, 1 μg/mL of LPS, or Tris-HCl (pH 8.0). For the positive control, ATP (4 mM) was added at the last 2 h of incubation. Six hours after the treatment, the cells were collected, and the cell lysis was analyzed by western blotting. In all groups, n = 3. Experiments were repeated three times. **(D)** Release of IL-1β and IL-18 in the supernatant of *Eh*-rPrx-treated cells. RAW264.7 cells were pretreated with TAK242 (1 μM) for 1 h or oATP (500 μM) for 2 h. Next, cells were treated with either 5 μg/mL of *Eh*-rPrx, 1 μg/mL of LPS, or Tris-HCl (pH 8.0). For the positive control, ATP (4 mM) was added at the last 2 h of incubation. Cell culture supernatant was collected after 12 and 24 h of treatment, and analyzed by enzyme-linked immunosorbent assay. In all groups, n=3. Experiments were repeated three times. Statistical analysis was conducted by Student’s *t*-test, and data are presented as mean ± SEM. *p < 0.05, **p < 0.01, ***p < 0.001, ns, not significant.

We further constructed recombinant *E. histolytica* Prx (*Eh*-rPrx) (XP_648522.1) and detected the endotoxin level of rPrx by tachypleus amebocyte lysate. The results showed that the endotoxin concentration of 500 μg/ml recombinant protein was lower than 0.5 EU/mL, meeting the national standard for medical products in the People’s Republic of China (GB/t14233.2-2005). Female C57BL/6 mice were treated with *Eh*-rPrx through intraperitoneal injection (100 μg/mouse). After 12 h of *Eh*-rPrx treatment, PEC (containing macrophages) were collected from the mice and NLRP3 inflammasome-related gene expression was assessed by qPCR. The expressions of *NLRP3*, *IL-1β*, and *caspase-11* were significantly increased (p < 0.05), while *IL-18* expression was only slightly increased (**Figure 3A**). Hence, priming of NLRP3 inflammasome in macrophages might be induced by *Eh*-rPrx.

### *Eh*-rPrx Activated NLRP3 Inflammasome Through Recognizing TLR4 Receptor

To further confirm the capability of *Eh*-rPrx to activate NLRP3 inflammasome, the expressions of NLRP3 inflammasome-related genes following *Eh*-rPrx and RAW264.7 cell co-incubation were analyzed. The expression levels of *NLRP3*, *IL-1β*, *IL-18*, and *caspase-11* were significantly increased after 6 h (p < 0.05) (**Figure 3B**). In order to exclude the influence of His-tag and endotoxin on the recombinant protein, the other unrelated recombinant proteins including Igl-C and Hgl-A were used in this experiment. Igl-C and Hgl-A are fragments of Igl and Hgl, respectively, which are parts of Gal/GalNAc lectin from *E. histolytica*. These recombinant proteins were obtained by the same method with Eh-rPrx, and were co-incubated with RAW264.7 cells. The results showed that the other recombinant proteins could only induce weak expression of *NLRP3* gene, which was significantly different from that found by *Eh*-rPrx (P < 0.05) (**Supplementary Figure 3A**). Following pretreatment with different dilutions of 4G6 ascites, the ability of *Eh*-rPrx in priming NLRP3 inflammasome was inhibited in a concentration-dependent manner (**Supplementary Figure 3B**).

The further study confirmed that TLR4 inhibitor TAK242 and P2X7 receptor inhibitor oATP significantly inhibited the ability of *Eh*-Prx to prime NLRP3 inflammasome, the protein levels of NLRP3 and pro-caspase-11 were significantly increased after incubation for 6 h (p < 0.05), which were then significantly inhibited by TAK242 (p < 0.05) (**Figure 3C**). Cleaved caspase-1 was detected 24 h after *Eh*-rPrx treatment. Compared with the negative control, the secretion level of cleaved caspase-1 in *Eh*-rPrx was increased, which was further inhibited by either TAK242 or oATP (**Supplementary Figure 4**). Additionally, IL-1β and IL-18 secretion was significantly increased at 24 h of incubation, while such an effect was inhibited by either TAK242 or oATP (p < 0.05) (**Figure 3D**).

**Figure 4:**
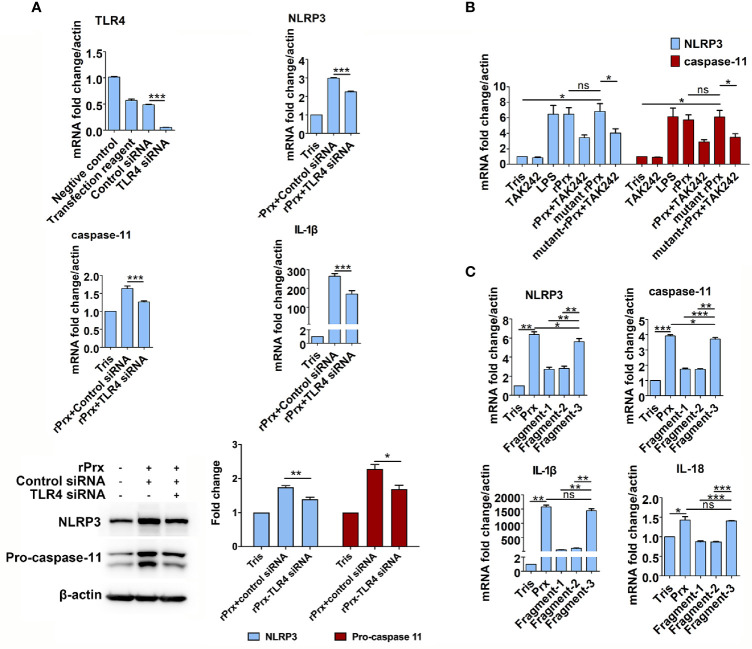
Mechanism of *Eh*-rPrx recognition *via* TLR4 receptor. **(A)** *Eh*-rPrx recognition through TLR4 receptor was confirmed *via* TLR4 siRNA. RAW264.7 cells were treated with control siRNA (50 nM) and TLR4 siRNA (50 nM) for 24 h. Next, the supernatant was removed, and the cells were treated with *Eh*-rPrx (5 μg/mL) for 6 h. Expression levels of NLRP3-related genes and proteins were detected by qPCR and w estern blotting, respectively. In all groups, n = 3. Experiments were repeated three times. **(B)** Activation of NLRP3 Inflammasome independently of Prx’s reductase activity. *Eh*-rPrx functioned independently of antioxidant activity. For detection of NLRP3-related gene expression levels, RAW264.7 cells were pretreated with TLR4 inhibitor TAK242 (1 μM) for 1 h, prior to 5 μg/mL of *Eh*-rPrx or mutant *Eh*-rPrx. LPS (1 μg/mL) and Tris-HCl (pH 8.0) were used as the positive and negative controls, respectively. Six hours after the treatment, the expression levels of related genes were measured by qPCR and shown as 2-ΔΔCt of the target gene relative to β-actin, normalized with corresponding values in the negative control. In all groups, n = 3. Experiments were repeated three times. **(C)** The C-terminal of *Eh*-Prx was identified as the functional domain responsible for activating NRLP3 inflammasome. Three fragments of *Eh*-Prx were constructed; fragment-1 to fragment-3 each contained 100 amino acids at the N-terminal, middle segment, and C-terminal. Following incubation of the fragments and RAW264.7 cells for 6 h, the expression levels of related genes were measured by qPCR and shown as 2-ΔΔCt of the target gene relative to β-actin, normalized with corresponding values in the negative control. In all groups, n = 3. Experiments were repeated three times. Statistical analysis was conducted by Student’s *t*-test, and data are expressed as mean ± SEM. *p < 0.05, **p < 0.01, ***p < 0.001, ns, not significant.

The results mentioned above suggest that TLR4 is an important receptor through which *Eh*-rPrx activates NLRP3 inflammasome. Therefore, we used TLR4 siRNA for further validation. After 24 h of treatment, TLR4 siRNA could significantly disrupt TLR4 mRNA in macrophages (p < 0.001) (**Figure 4A**). Following incubating the TLR4 siRNA-treated cells with *Eh*-rPrx, the gene expression levels of *NLRP3*, *IL-1β*, and *caspase-11* were significantly down-regulated (p < 0.05) (**Figure 4A**), and the protein levels of NLRP3 and caspase-11 were also significantly reduced (p < 0.05) (**Figure 4A**).

### Priming of NLRP3 Inflammasome Independent of Prx’s Reductase Activity

To investigate if the *Eh*-rPrx-induced priming of NLRP3 inflammasome was dependent on reductase activity, a mutant *Eh*-rPrx was constructed *via* the transformation of cysteine at two cysteine active sites from TGT to GGT (23). The antioxidant activity of the recombinant proteins was detected using the metal-catalyzed oxidation (MCO) system, which indicated that 5 μM of *Eh*-rPrx was sufficient to protect the superhelix plasmid from oxidative damage caused by free radicals (**Supplementary Figure 5A**), while the antioxidant activity of the mutant *Eh*-rPrx was dramatically decreased (**Supplementary Figure 5B**). However, the mutant *Eh*-rPrx still showed a potent ability to induce the expressions of *NLRP3* and *caspase-11* following incubating with RAW264.7 cells, which was consistent with that found in non-mutant *Eh*-rPrx (**Figure 4B**).

Further, to investigate the specific domain of *Eh*-rPrx in priming NLRP3 inflammasome, three fragments of *Eh*-Prx were constructed in this study. The endotoxin levels of these proteins were detected by tachypleus amebocyte lysate as mentioned above. The results showed that the endotoxin concentration of 500 μg/ml recombinant protein was lower than 0.5 EU/mL, meeting the national standard for medical products in the People’s Republic of China (GB/t14233.2-2005). Following treating RAW264.7 cells with the three fragments separately, Fragment-3 (C-terminal) was found to significantly induce the expression of NLRP3 inflammasome-related genes (p < 0.01), which was comparable to levels induced by full-length Prx (**Figure 4C**).

### Inhibition of TLR4 Receptor Blocked Apoptosis and Mitochondrial Oxidative Damage Induced by Prx

**Figure 5:**
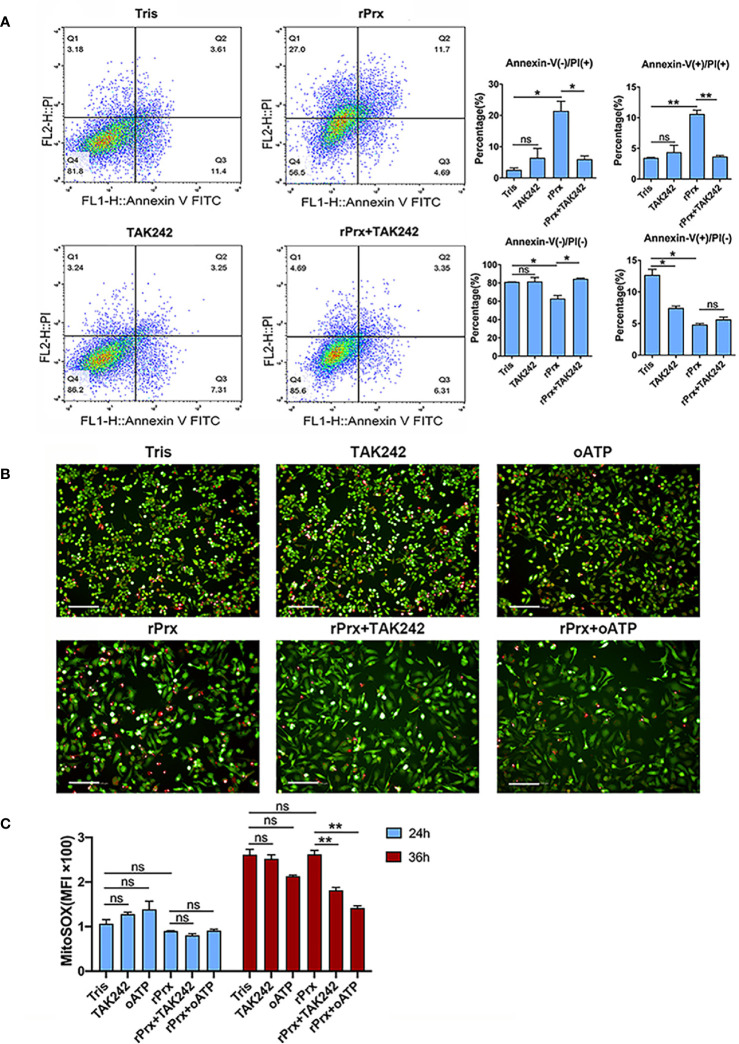
Inhibition of TLR4 receptor blocked apoptosis and mitochondrial oxidative damage induced by Prx. **(A)** Following pretreatment with TAK242 (1 μM) for 1 h, RAW264.7 cells were incubated with *Eh*-rPrx (5 μg/ml) for 24 h, then suspended in 500 μL of 1× binding buffer (Sigma-Aldrich, APOAF) at a concentration of 10^6^ cells/ml. Following incubation at room temperature for 10 min, 5 μL of Annexin V FITC conjugate (Sigma-Aldrich, APOAF) and 10 μL of propidium iodide solution (Sigma-Aldrich, APOAF) were added to each suspension. Fluorescence of the cells was immediately determined by using a flow cytometer. In all groups, n = 3. Experiments were repeated three times. **(B)** Quantitative analysis of mitochondrial ROS MFI in RAW264.7 cells treated with rPrx. RAW264.7 cells were pretreated with TAK242 (1 μM) for 1 h or oATP (500 μM) for 2 h. Next, the cells were treated with 5 μg/mL of *Eh*-rPrx or Tris-HCl (pH 8.0) as the negative control. After 24 h or 36 h of treatment, the live cells were stained with MitoSOX (5 μM) for 10 min, and then with CFSE (2 μM) for counterstaining. The cells were immediately observed and quantified through high-content screening analysis. In all groups, n = 3. Experiments were repeated three times. **(C)** The fluorescence images of RAW264.7 cells after 36 h of rPrx treatment. Red fluorescence, MitoSOX™ Red; green fluorescence, CFSE; scale bar, 100 μm. In all groups, n = 3. Experiments were repeated three times. Statistical analysis was conducted by Student’s *t*-test, and data are expressed as mean ± SEM. *p < 0.05, **p < 0.01, ns, not significant.

To investigate if the *Eh*-rPrx-induced NLRP3 inflammasome activation was accompanied through cell death, apoptosis was assessed following *Eh*-rPrx and RAW264.7 cells co-incubation. After 24 h of treatment, we observed that *Eh*-rPrx-induced activation of NLRP3 inflammasome was accompanied by a significant degree of apoptosis, shown by the higher proportion of PI-positive cells in the *Eh*-rPrx group than those in the negative control group (**Figure 5A**). Furthermore, *Eh*-rPrx-induced apoptosis was significantly inhibited by the TLR4 receptor inhibitor TAK242 (p < 0.05) (**Figure 5A**).

MitoSOX was used to further detect the levels of mitochondrial ROS in live RAW264.7 cells incubated with *Eh*-rPrx. After 24 h of incubation, *Eh*-rPrx unexpectedly induced slightly decreased mitochondrial ROS Mean Fluorescence Intensity (MFI), in comparison with the negative control, while the decrease was not significant (**Figure 5B**). After 36 h of treatment, *Eh*-rPrx caused a slight increase of ROS (**Figure 5B**), which was significantly inhibited by TAK242 and oATP (p < 0.01) (**Figures 5B, C**). However, the ROS levels in both TAK242 and oATP groups were insignificantly different from that in the negative control (**Figures 5B, C**). We speculated that the mitochondrial ROS level up-regulated by *Eh*-rPrx was mitigated by other signaling pathway activated by *Eh*-rPrx.

## Discussion

Over the last few decades, evidence has suggested that intestinal invasion by trophozoites is primarily attributed to the compromised intestinal barrier integrity and intestinal inflammation during infection. Intestinal epithelium is an important barrier to prevent *E. histolytica* trophozoite. To survive in human intestine, various virulence factors of *E. histolytica*, such as Gal/GalNAc lectin, peroxiredoxin, cysteine protease, etc., contribute to the colonization of this trophozoite on the mucus layer and the resistance towards immunizing inflammatory reaction (25). Gal/GalNAc lectin could bind with the receptors of colonic epithelial cells, erythrocytes, and innate host immune cells such as neutrophils and macrophages, and triggers strong pro-inflammatory responses through promoting the release of pro-inflammatory cytokines such as IL-1β, TNF-α, IL-12, IL-6, and nitric oxide (NO) (26). Intestinal epithelial cells are intact at the early stages of inflammation; the binding of Gal/GalNAc lectin to colonic epithelial cells allows surface bound EhCP5 to ligate αvβ3 integrin that leads to NF-κB activation and pro-inflammatory responses. Then, CP5 from *E. histolytica* activates extracellular pro-IL-1β which, in addition to the chemoattractant secreted by epithelial cells, recruits neutrophils and macrophages, resulting in a serious inflammatory reaction (27). Therefore, a series of *E. histolytica*-secreted molecules bind to PRRs on immune cells in the lamina propria of the intestine, activating the inflammatory pathway (9). Peroxiredoxin that has potent antioxidant activity localized in the cytoplasm of trophozoites can protect *E. histolytica* from ROS-induced damage (15, 28).

Although chronic inflammation and macrophages are considered important in *E. histolytica* infection, large amounts of NO produced by macrophages, which synthesize highly oxidizing ONOO^-^, along with reactive oxygen intermediates (e.g., ${\text{O}}_{2}^{-}$ ), are considered responsible for host tissue destruction and invasiveness. In this study, we revealed that the trophozoites could upregulate Prxs expression and abundantly secret Prxs when encountering host cells including macrophages. We found that the intracellular amount of Prx in trophozoites was significantly increased after the co-incubation of trophozoites with RAW264.7 or CHO cells. Previous *in vivo* studies showed that Prxs could be associated with *E. histolytica* pathogenicity, without disclosing the mechanisms involved in the regulation of parasite virulence (2, 29). Moreover, the Prxs of several parasites have been found to act as PAMPs through PRRs on macrophages (21–23). Although Prxs of *E. histolytica* are associated with ALA (18, 30), whether they serve as PAMPs and activate macrophages has not been examined. Nevertheless, a series of molecules secreted by *E. histolytica* were confirmed as the activators of PRRs on macrophages. For instance, *E. histolytica* lipophosphopeptidogylcan could activate submucosal macrophages through TLR2 and TLR4 receptors (31); Gal/GalNAc lectin could upregulate gene expressions of NF-κB and mitogen-activated protein kinase signaling pathways in macrophages (32, 33). Furthermore, recent studies have indicated that inflammasome in macrophages could be activated in a contact-dependent manner *via* live *E. histolytica* trophozoites, suggesting the importance of Gal/GalNAc lectin adhesion for trophozoite pathogenicity (8, 34). Moreover, *E. histolytica* CP5 was identified as a contact-dependent inducer for the NLRP3 inflammasome (6); however, proteins other than CP5 that activate the NLRP3 inflammasome have not yet been previously reported.

Herein, our data showed that the extensive distribution of *Eh*-Prx in ALA tissue and the coincidently overlapped distribution areas of Prxs and macrophage aggregation sites. The immunohistochemical staining of IL-1β in ALA tissue indicated that inflammasome might be activated at the common site where Prx and macrophages are distributed. Moreover, qPCR results indicated that NLRP3 inflammasome was primed in ALA tissue. Considering that NLRP3 is mainly expressed in Kupffer cells (35), we speculated that NLRP3 inflammasome in macrophages was activated during trophozoites invasion. Following incubating trophozoites with RAW264.7 or CHO-K1 cells, we detected that the Prx protein expression level in trophozoites was up-regulated. Our study indicated that trophozoites secret abundant Prx when contacting with host cells, and Prx of *E. histolytica* could play stable and durable contact-independent effects on macrophages. More importantly, no Prx secretion was detected in unstimulated trophozoites. Moreover, both native and recombinant Prxs were confirmed to prime NLRP3 inflammasome in macrophages *via* TLR4 receptor. Therefore, a large amount of Prx are released when trophozoites invade host’s tissues, while the secreted Prx can induce NLRP3 inflammasome in macrophages in a contact-independent manner.

Previous researches have reported that *E. histolytica* activates macrophage inflammasome in a contact-dependent manner (6, 8, 34, 36) and *Eh*CP-A5 was the key molecule (34). Our study indicated that NLRP3 inflammasome could be activated *via Eh*-rPrx alone, accompanied with the activation of caspase-1 and significant secretion of IL-1β and IL-18, while it was significantly inhibited by TAK242 or oATP, suggesting that *Eh*-rPrx activates the NLRP3 inflammasome in a contact-independent manner through canonical pathway.

Although most *Eh-*Prx studies focus on its antioxidant activity, our results demonstrated that *Eh*-rPrx-induced activation of the NLRP3 inflammasome was independent of the antioxidant activity, indicating that *E. histolytica* has a stable and durable effect on macrophages following tissue or organ invasion. Not coincidentally, helminth Prxs, without antioxidant activity, were also capable of inducing macrophage polarization (23). Among the three *Eh*-rPrx fragments investigated, the 100 amino acids at the C-terminal (Fragment-3) had comparable effects as the intact protein, indicating that the C-terminal of *Eh*-rPrx is crucial for activating the NLRP3 inflammasome in macrophages. Interestingly, our previous work demonstrated that the C-terminal also serves as the recognition site of mAb 4G6 (37). Results above suggested the negligible function of residual endotoxin in recombinant Prx.

The NLRP3 inflammasome can be activated by various stimuli, among which mitochondrial ROS and mitochondrial DNA, released *via* oxidative damage, are relevant to NLRP3 inflammasome activation (38). Mitochondrial autophagy clears the damaged or dysfunctional mitochondria, serving as a regulatory mechanism of NLRP3 inflammasome activation (38). Furthermore, our data suggest that *Eh*-rPrx was speculated to induce oxidative damage on mitochondria, while other cellular pathways (e.g., autophagy) activated by *Eh*-rPrx can clear the damaged mitochondria, which is also supported by our previous work (37).

In summary, this study identified a new mechanism of *E. histolytica*-induced chronic inflammation. To the best of our knowledge, this is the first study to describe the C-terminal of *Eh*-rPrx as the key domain responsible for activating NLRP3 inflammasome through recognizing TLR4 receptor in macrophages. Thus, the C-terminal of *Eh*-rPrx is a potential drug target for treating *E. histolytica* infection. Importantly, the present study provides evidence to support that TLR4-binding domain of Prx from *E. histolytica* can activate NLRP3 inflammasome in a contact-independent manner.

## Data Availability Statement

The raw data supporting the conclusions of this article will be made available by the authors, without undue reservation.

## Ethics Statement

The animal study was reviewed and approved by Institutional Animal Care and Use Committee of School of Basic Medical Sciences, Fudan University.

## Author Contributions

Experiment design, XC and XL. Experiments, XL, MF, YQZ, YHZ, RZ, HZ, ZP, and XC. Data analysis and scientific discussion, XL, MF, HT, and XC. Writing manuscript, XL, MF, and XC. All authors contributed to the article and approved the submitted version.

## Funding

This work was supported by the National Key Research and Development Program of China (2018YFA0507304) (http://service.most.gov.cn/) and National Natural Science Foundation of China (81630057) (http://www.nsfc.gov.cn/) to XC.

## Conflict of Interest

The authors declare that the research was conducted in the absence of any commercial or financial relationships that could be construed as a potential conflict of interest.

## Publisher’s Note

All claims expressed in this article are solely those of the authors and do not necessarily represent those of their affiliated organizations, or those of the publisher, the editors and the reviewers. Any product that may be evaluated in this article, or claim that may be made by its manufacturer, is not guaranteed or endorsed by the publisher.
