# Supplementary material for: A Novel TLR4-Binding Domain of Peroxiredoxin From Entamoeba histolytica Triggers NLRP3 Inflammasome Activation in Macrophages
Source: Front Immunol. 2021 Sep 30;12:758451. doi: 10.3389/fimmu.2021.758451 (PMC8515043; doi:10.3389/fimmu.2021.758451)
Supplement: Supplementary file 1 [file DataSheet_1.docx]

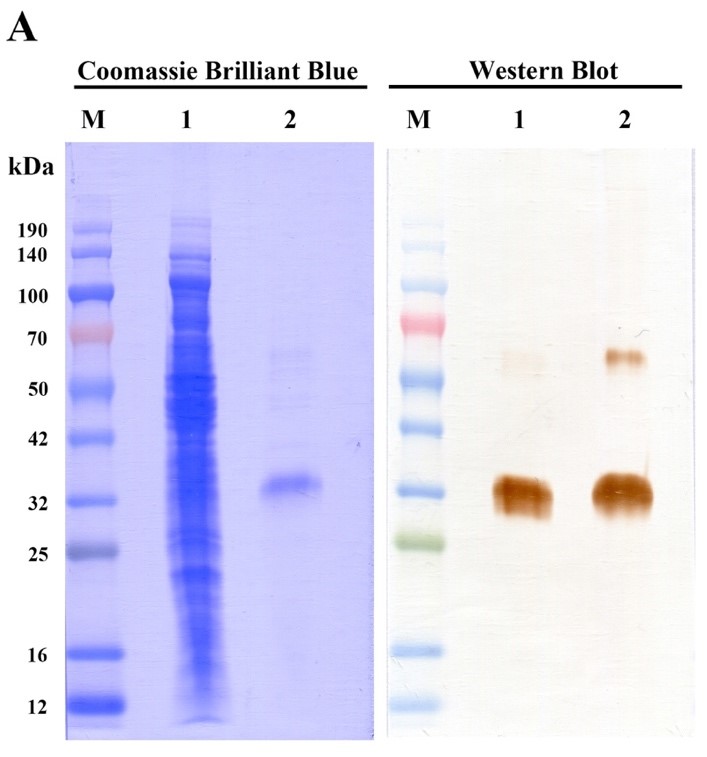


**Supplement Figure 1.** The purified native Prx was identified by Coomassie brilliant blue and WB. M, marker; 1, crude lysate; 2, purified protein.


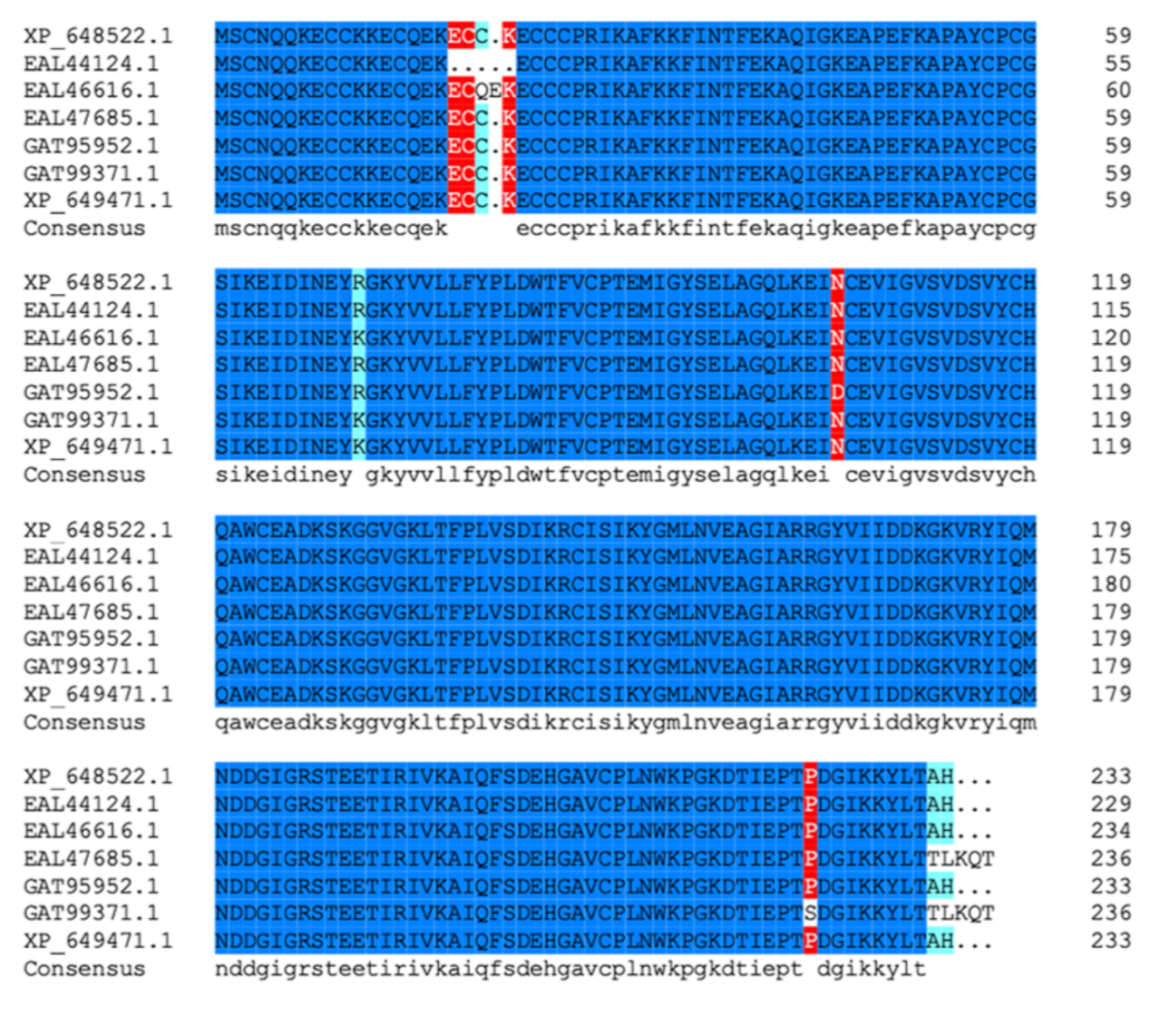


**Supplement Figure 2.** Sequence alignment of native Prx using DNAMAN 6.0. The consistency of amino acid residues among proteins was 97.54%.


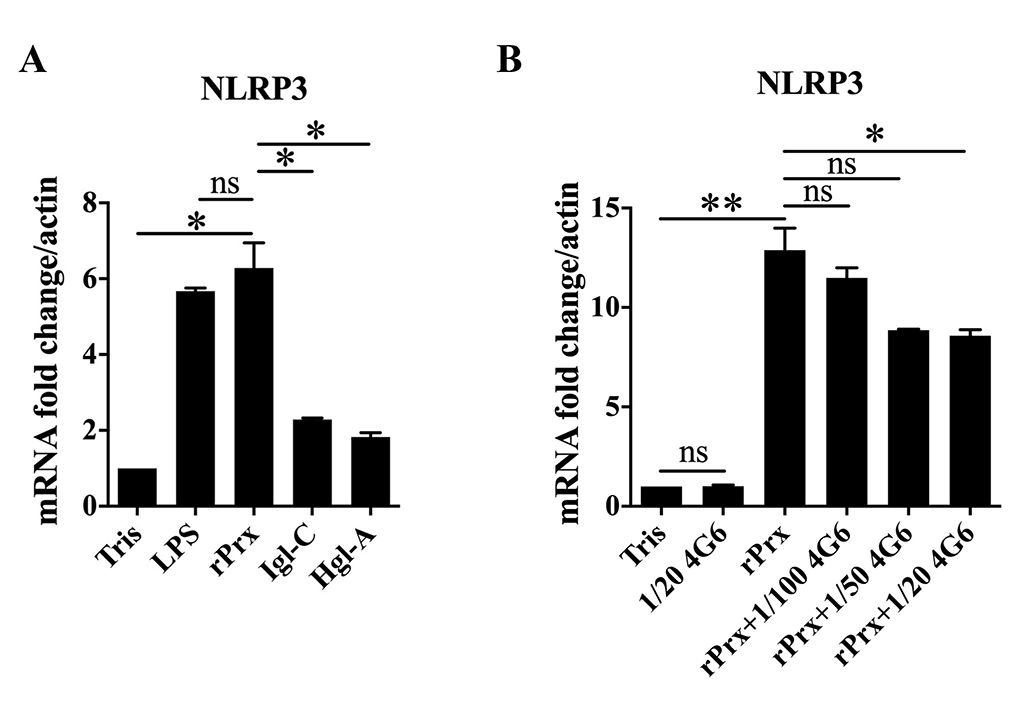


**Supplement Figure 3.** The effect of His-tag and endotoxin in rPrx on NLRP3 gene expression. **(A)** The effect of recombinant proteins on expression of *NLRP3* gene was detected by qPCR. All the recombinant proteins were obtained from E. coli. The final concentration was 5 μg/mL. After treatment of RAW264.7 cells with proteins for 6 h, Cells were harvested and gene expression levels of related proteins measured using qPCR; shown as 2-ΔΔCt of the target gene relative to β-actin; normalized with corresponding values in the negative control. **(B)** The effect of 4G6 on expression of *NLRP3* gene by rPrx. After pretreated with different dilutions of 4G6 ascites, rPrx was incubated with RAW264.7 cells for 6 h. Cells were harvested and gene expression levels of related proteins measured using qPCR; shown as 2-ΔΔCt of the target gene relative to β-actin; normalized with corresponding values in the negative control. Statistical analysis using Student’s *t*-test, mean ± SEM (n=3). *p<0.05, **p<0.01, ns, not significant.


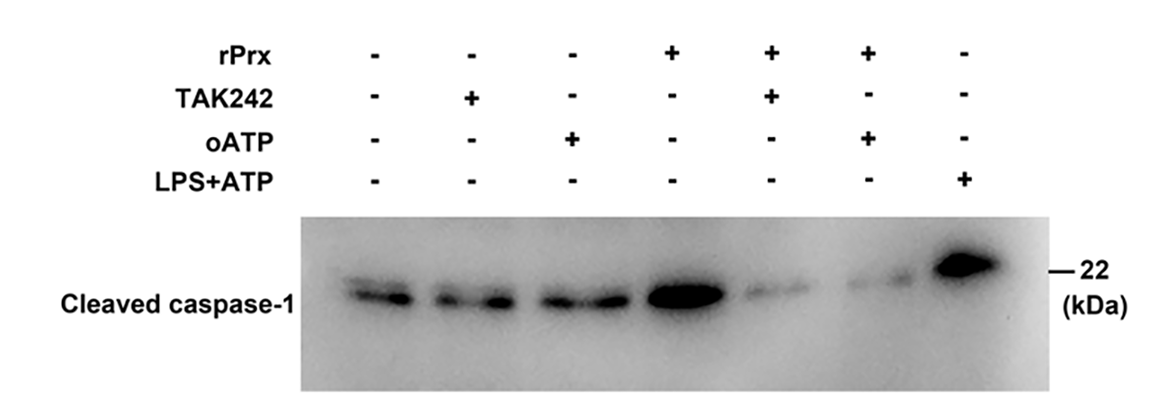


**Supplement Figure 4.** Cleaved caspase-1 in RAW264.7 cell supernatant was detected by western blotting. After co-incubation of rPrx and RAW264.7 cells for 24 h, the cell supernatant was collected. Cold acetone precipitation was conducted to extract protein from the cultural supernatant. Afterwards, protein from the same volume of supernatant was analyzed by western blotting.



 **Supplement Figure 5.** Confirmation of *Eh*-rPrx antioxidant activity. A mixture containing supercoiled pUC19 plasmid DNA (500 ng/lane), components of the MCO system (1.5 μM FeCl3 and 0.5 mM DTT), and various concentration of *Eh*-rPrx or mutant *Eh*-rPrx was incubated for 30 min, after pre-incubation without a plasmid for 5 min, and then electrophoresed on a 0.8% agarose gel.
