# Supplementary material for: A Novel TLR4-Binding Domain of Peroxiredoxin From Entamoeba histolytica Triggers NLRP3 Inflammasome Activation in Macrophages
Source: Front Immunol. 2021 Sep 30;12:758451. doi: 10.3389/fimmu.2021.758451 (PMC8515043; doi:10.3389/fimmu.2021.758451)
Supplement: Supplementary file 2 [file Table_1.docx]

| **Supplementary Table 1** Analysis of native Prx by Mass Spectrometry | | | |
| --- | --- | --- | --- |
| Accession | Description | Sum PEP Score | # AAs |
| XP_649471.1 | peroxiredoxin, putative [*Entamoeba* *histolytica* HM-1:IMSS] | 252.647 | 233 |
| EAL46616.1 | peroxiredoxin [*Entamoeba histolytica* HM-1:IMSS] | 250.737 | 234 |
| EAL47685.1 | peroxiredoxin, putative [*Entamoeba* *histolytica* HM-1:IMSS] | 241.321 | 237 |
| GAT99371.1 | peroxiredoxin [*Entamoeba histolytica*] | 240.76 | 237 |
| XP_648522.1 | peroxiredoxin [*Entamoeba histolytica* HM-1:IMSS] | 239.361 | 233 |
| EAL44124.1 | peroxiredoxin [*Entamoeba histolytica* HM-1:IMSS] | 237.451 | 229 |
| GAT95952.1 | peroxiredoxin [*Entamoeba histolytica*] | 229.873 | 233 |
| EMH74380.1 | clathrin heavy chain, putative [Entamoeba histolytica HM-1:IMSS-B] | 131.058 | 1704 |
| 3QTP | A Chain A, Enolase 1 | 125.611 | 441 |
| BAA20970.1 | antigen, partial [*Entamoeba histolytica*] | 120.76 | 133 |
| BAN40050.1 | glyceraldehyde-3-phosphate dehydrogenase, putative [*Entamoeba* *histolytica*] | 114.595 | 334 |
| BAN39768.1 | glyceraldehyde-3-phosphate dehydrogenase, putative [*Entamoeba* *histolytica*] | 114.339 | 334 |
| BAN38965.1 | actin, putative [*Entamoeba histolytica*] | 110.333 | 376 |
| EMD47007.1 | alkyl hydroperoxide reductase, putative [*Entamoeba histolytica* KU27] | 105.686 | 81 |
| BAN38519.1 | actin, putative [*Entamoeba histolytica*] | 102.914 | 376 |
| BAA20964.1 | antigen, partial [*Entamoeba histolytica*] | 99.42 | 133 |
| AAB49653.1 | pyruvate:ferredoxin oxidoreductase [*Entamoeba histolytica*] | 98.956 | 1162 |
| AAA29102.1 | heat shock protein 70, hsp70A2 [*Entamoeba histolytica*] | 88.677 | 656 |
| EMS16513.1 | nuclease domain containing protein, putative [*Entamoeba histolytica* HM-3:IMSS] | 85.231 | 861 |
| BAN38227.1 | 60S ribosomal protein L7a, putative [*Entamoeba histolytica*] | 83.051 | 286 |
| EMS13483.1 | peroxiredoxin, putative [*Entamoeba* *histolytica* HM-3:IMSS] | 78.639 | 81 |
| GAT93723.1 | isoleucyl-tRNA synthetase putative [*Entamoeba histolytica*] | 77.365 | 1056 |
| EMD47757.1 | hsp70 BiP, putative [*Entamoeba histolytica* KU27] | 75.642 | 661 |
| Q24803.1 | RecName: Full=Aldehyde-alcohol dehydrogenase 2; Includes: RecName: Full=Alcohol dehydrogenase; Short=ADH; Includes: RecName: Full=Acetaldehyde dehydrogenase; Short=ACDH | 74.954 | 870 |
| BAN38170.1 | ribosomal protein S4, putative [*Entamoeba histolytica*] | 74.613 | 326 |
| EAL50431.2 | alcohol dehydrogenase, putative [*Entamoeba histolytica* HM-1:IMSS] | 74.594 | 870 |
| BAN37516.1 | 60S ribosomal protein L4, putative [*Entamoeba histolytica*] | 68.938 | 431 |
| EAL50904.1 | acetyl-CoA synthetase, putative [*Entamoeba histolytica* HM-1:IMSS] | 67.923 | 713 |
| P31018.1 | RecName: Full=Elongation factor 1-alpha; Short=EF-1-alpha | 64.562 | 430 |
| EMD47533.1 | coatomer subunit beta'3, putative [*Entamoeba histolytica* KU27] | 64.074 | 795 |
| BAN37555.1 | elongation factor 1-alpha 1 [*Entamoeba histolytica*] | 63.989 | 442 |
| EMD46770.1 | Hypothetical protein EHI5A_005960 [*Entamoeba histolytica* KU27] | 63.947 | 559 |
| EMD44412.1 | 60S ribosomal protein L5 [*Entamoeba* *histolytica* KU27] | 63.395 | 286 |
| GAT94658.1 | gal galnac lectin subunit igl1 [*Entamoeba histolytica*] | 62.842 | 1101 |
| BAN37465.1 | 14-3-3 protein 3 [*Entamoeba* *histolytica*] | 61.452 | 240 |
| GAT97819.1 | Ras family GTPase [*Entamoeba histolytica*] | 59.838 | 184 |
| BAN39620.1 | sulfate adenylyltransferase, putative [*Entamoeba histolytica*] | 56.807 | 477 |
| BAN37506.1 | 60S ribosomal protein L4, putative [*Entamoeba histolytica*] | 55.893 | 431 |
| BAN38513.1 | 60S ribosomal protein L3, putative [*Entamoeba histolytica*] | 54.635 | 402 |
| BAN37611.1 | 40S ribosomal protein S9 [*Entamoeba* *histolytica*] | 52.537 | 185 |
| BAN37647.1 | methionine aminopeptidase, putative [*Entamoeba histolytica*] | 52.264 | 413 |
| EMD49122.1 | dTDPD-glucose 4,6-dehydratase, putative [*Entamoeba histolytica* KU27] | 51.068 | 365 |
| XP_657379.1 | histone acetyltransferase, putative [*Entamoeba histolytica* HM-1:IMSS] | 50.602 | 576 |
| ENY66034.1 | 60S ribosomal protein L27, putative [*Entamoeba histolytica* HM-1:IMSS-A] | 50.405 | 139 |
| EAL45913.2 | phosphoribulokinase/uridine kinase family protein [*Entamoeba histolytica* HM-1:IMSS] | 50.33 | 393 |
| BAN38036.1 | actin-like protein, putative [*Entamoeba histolytica*] | 48.979 | 401 |
| EMD42382.1 | adapter-related protein complex 3 (AP-3) subunit, putative [*Entamoeba histolytica* KU27] | 48.83 | 1030 |
| GAT95246.1 | adapter-related protein complex 3 p 3 subunit putative [*Entamoeba* *histolytica*] | 48.685 | 1030 |
| BAN37715.1 | 40S ribosomal protein SA, putative [*Entamoeba histolytica*] | 48.609 | 254 |
| EMD48002.1 | glycerol3-phosphate dehydrogenase, putative [*Entamoeba histolytica* KU27] | 48.474 | 1069 |
| EMS12313.1 | 60S ribosomal protein L27, putative [*Entamoeba histolytica* HM-3:IMSS] | 48.258 | 139 |
| BAN37898.1 | EF-hand calcium-binding domain containing protein [*Entamoeba* *histolytica*] | 48.245 | 227 |
| BAN37662.1 | fructose-1,6-bisphosphate aldolase, putative [*Entamoeba histolytica*] | 47.922 | 330 |
| XP_651009.2 | elongation factor 2 [*Entamoeba* *histolytica* HM-1:IMSS] | 47.914 | 841 |
| AAG09783.1 | Fe-hydrogenase [*Entamoeba histolytica*] | 46.878 | 468 |
| GAT92249.1 | Fe-s cluster assembly protein nifu putative [*Entamoeba histolytica*] | 46.521 | 348 |
| EAL47172.1 | aminopeptidase, putative [*Entamoeba histolytica* HM-1:IMSS] | 44.975 | 827 |
| EAL52045.2 | hypothetical protein EHI_151220 [*Entamoeba histolytica* HM-1:IMSS] | 44.403 | 759 |
| EAL51127.1 | PIWI, putative [*Entamoeba histolytica* HM-1:IMSS] | 44.141 | 937 |
| EAL52137.1 | Ran family GTPase [*Entamoeba* *histolytica* HM-1:IMSS] | 44.071 | 219 |
| 4Y0V | B Chain B, ADP-ribosylation factor 1 | 43.71 | 178 |
| AAF32317.1 | Rab7-like GTPase, partial [*Entamoeba histolytica*] | 43.563 | 206 |
| BAN37676.1 | 40S ribosomal protein S3, putative [*Entamoeba histolytica*] | 43.326 | 244 |
| EMH72353.1 | Rab family GTPase [*Entamoeba histolytica* HM-1:IMSS-B] | 42.474 | 208 |
| XP_648378.2 | hypothetical protein EHI_164470 [*Entamoeba histolytica* HM-1:IMSS] | 42.466 | 807 |
| O76321.1 | RecName: Full=Rho-related protein racG; Flags: Precursor | 42.108 | 199 |
| EMS12768.1 | ribosomal protein S13p/S18e, putative [*Entamoeba histolytica* HM-3:IMSS] | 41.405 | 263 |
| GAT98466.1 | clathrin coat assembly protein putative [*Entamoeba histolytica*] | 40.882 | 414 |
| ENY64343.1 | inorganic pyrophosphatase, putative [*Entamoeba histolytica* HM-1:IMSS-A] | 40.703 | 244 |
| EMD49035.1 | leucinerich repeat-containing protein [*Entamoeba histolytica* KU27] | 40.621 | 883 |
| BAN37587.1 | 40S ribosomal protein S7, putative [*Entamoeba histolytica*] | 40.543 | 196 |
| BAA08651.1 | putative NADH dependent alcohol dehydrogenase [*Entamoeba histolytica*] | 40.07 | 395 |
| EAL45974.1 | phosphoribulokinase /uridine kinase family protein [*Entamoeba histolytica* HM-1:IMSS] | 39.798 | 543 |
| EAL47299.1 | 40S ribosomal protein S5, putative [*Entamoeba histolytica* HM-1:IMSS] | 39.247 | 205 |
| EMS14346.1 | 40S ribosomal protein S7, putative [*Entamoeba histolytic*a HM-3:IMSS] | 37.754 | 201 |
| ABS59295.1 | 30,000-Mr antigen, partial [*Entamoeba histolytica*] | 37.197 | 33 |
| BAN37726.1 | 40S ribosomal protein S2, putative [*Entamoeba histolytica*] | 36.61 | 255 |
| XP_653886.2 | heat shock protein70, hsp70A2, putative [*Entamoeba histolytica* HM-1:IMSS] | 35.109 | 665 |
| EMS15814.1 | Coronin, putative [*Entamoeba histolytica* HM-3:IMSS] | 34.986 | 457 |
| EMH73927.1 | 2,3-bisphosphoglycerate-independent phosphoglycerate mutase, putative [*Entamoeba histolytica* HM-1:IMSS-B] | 34.64 | 555 |
| BAN38088.1 | galactokinase, putative [*Entamoeba histolytica*] | 34.159 | 389 |
| BAN37773.1 | malic enzyme, putative [*Entamoeba histolytica*] | 33.815 | 487 |
| EMS15096.1 | 40S ribosomal protein S16, putative [*Entamoeba histolytica* HM-3:IMSS] | 33.148 | 225 |
| BAD82837.1 | small GTPase EhRab2C, partial [*Entamoeba histolytica*] | 32.958 | 231 |
| BAN39910.1 | 14-3-3 protein 2 [*Entamoeba histolytica*] | 32.695 | 238 |
| EMH72419.1 | acetyl-CoA carboxylase alpha subunit domain containing protein [*Entamoeba histolytica* HM-1:IMSS-B] | 32.515 | 1244 |
| ENY60046.1 | zinc finger in N-recognin protein, putative [*Entamoeba histolytica* HM-1:IMSS-A] | 31.98 | 1385 |
| XP_649335.1 | Rab family GTPase [*Entamoeba histolytica* HM-1:IMSS] | 31.922 | 213 |
| AAK69775.1 | mitochondrial carrier family protein [*Entamoeba histolytica*] | 31.922 | 276 |
| EMS11792.1 | 1,4-alpha-glucan-branching enzyme [*Entamoeba histolytica* HM-3:IMSS] | 31.458 | 680 |
| EMD46417.1 | 60S ribosomal protein L31 [*Entamoeba histolytica* KU27] | 31.452 | 150 |
| GAT92860.1 | 60S ribosomal protein L9 putative [*Entamoeba histolytica*] | 31.397 | 197 |
| AAC15759.1 | 19S cap proteasome S2 subunit [*Entamoeba histolytica*] | 31.093 | 843 |
| XP_653428.1 | protein phosphatase, putative [*Entamoeba histolytica* HM-1:IMSS] | 31.075 | 304 |
| ENY63152.1 | 40S ribosomal protein S15A, putative [*Entamoeba histolytica* HM-1:IMSS-A] | 31.071 | 130 |
| 3U40 | A Chain A, Purine nucleoside phosphorylase | 30.983 | 242 |
| EMD46043.1 | aminoacylhistidine dipeptidase, putative [*Entamoeba histolytica* KU27] | 30.708 | 516 |
| XP_655385.1 | thioredoxin, putative [*Entamoeba histolytica* HM-1:IMSS] | 30.184 | 127 |
| P32022.2 | RecName: Full=Galactose-inhibitable lectin 170 kDa subunit | 30.032 | 1278 |
